# Detection of Olfactory Dysfunction Using Olfactory Event Related Potentials in Young Patients with Multiple Sclerosis

**DOI:** 10.1371/journal.pone.0103151

**Published:** 2014-07-21

**Authors:** Fabrizia Caminiti, Simona De Salvo, Maria Cristina De Cola, Margherita Russo, Placido Bramanti, Silvia Marino, Rosella Ciurleo

**Affiliations:** 1 IRCCS Centro Neurolesi “Bonino-Pulejo”, Messina, Italy; 2 Department of Biomedical Sciences and Morphological and Functional Imaging, University of Messina, Messina, Italy; National Institutes of Health, United States of America

## Abstract

**Background:**

Several studies reported olfactory dysfunction in patients with multiple sclerosis. The estimate of the incidence of olfactory deficits in multiple sclerosis is uncertain; this may arise from different testing methods that may be influenced by patients' response bias and clinical, demographic and cognitive features.

**Aims:**

To evaluate objectively the olfactory function using Olfactory Event Related Potentials.

**Materials and Methods:**

We tested the olfactory function of 30 patients with relapsing remitting multiple sclerosis (mean age of 36.03±6.96 years) and of 30 age, sex and smoking–habit matched healthy controls by using olfactory potentials. A selective and controlled stimulation of the olfactory system to elicit the olfactory event related potentials was achieved by a computer-controlled olfactometer linked directly with electroencephalograph. Relationships between olfactory potential results and patients' clinical characteristics, such as gender, disability status score, disease-modifying therapy, and disease duration, were evaluated.

**Results:**

Seven of 30 patients did not show olfactory event related potentials. Sixteen of remaining 23 patients had a mean value of amplitude significantly lower than control group (p<0.01). The presence/absence of olfactory event related potentials was associated with dichotomous expanded disability status scale (p = 0.0433), as well as inversely correlated with the disease duration (r = −0.3641, p = 0.0479).

**Conclusion:**

Unbiased olfactory dysfunction of different severity found in multiple sclerosis patients suggests an organic impairment which could be related to neuroinflammatory and/or neurodegenerative processes of olfactory networks, supporting the recent findings on neurophysiopathology of disease.

## Introduction

Olfactory dysfunction is often an early important manifestation of neurodegenerative diseases, such as Parkinson's disease [1]–[4], Alzheimer's disease [5]–[6], Huntington's disease [7], motor neuron disease [8], and its evaluation can be useful for diagnosis. The olfactory impairment in Multiple Sclerosis (MS) has also been reported [9]–[11], but to date, there are considerable disputes about. Moreover, it remains unclear whether olfactory loss occurs as an early symptom of MS. It has been suggested that some neurotropic viruses are involved in the development of neurodegenerative diseases. The olfactory loss in MS seems to be associated with the passage of human herpervirus-6 (HHV-6) into the central nervous system (CNS). Recently, HHV-6 has been detected in the olfactory bulbs and tracts and frequently in the nasal cavities of MS patients, supporting the hypothesis that this virus uses olfactory pathways as a route to enter into the CNS and then trigger the MS [12].

The incidence of olfactory dysfunction in MS is highly variable. Originally, it has been reported that the olfactory pathways (nerves and tracts) were spared in MS [13], with an estimation of incidence of olfactory changes by 1% [14]. Later, trials investigating the olfactory function in MS patients reported that these subjects had worse performances of 15% [15], 22% [16] and 38.5% [17] than healthy controls. This variability may arise from differences related to olfactory function testing methods and/or in study design, but it can also be related to corticosteroid treatment and to different MS subtypes. The smell function may improve during periods of disease remission and worsen during relapses. In addition, the olfactory dysfunction may also be an early indicator of disease progression in MS [18]. Several studies showed in MS patients a deterioration in the ability to detect odor threshold as well as in the ability to identify and discriminate the odors. Moreover, it has been suggested that the threshold detection is impaired in the early stage of disease, whereas the ability to identify and discriminate the odors is related to disability progression which is often associated with a cognitive impairment [11], [19]. Other authors highlighted that olfactory dysfunction in MS is related to the number of active plaques in the frontal and temporal lobes [9], [17], [19], [20], symptoms of anxiety and depression [9] and disability progression [7].

The studies evaluating the relationship between MS and the three olfactory abilities used psychophysical tests [11], [17], [19]–[23], such as the Sniffin' Sticks Test [24], [25] and the University of Pennsylvania Smell Identification Test (UPSIT) [26]. However, the psychophysical tests of identification and discrimination require complex cognitive functions and high attention levels which could be compromised also in the early stage of MS [27], [28].

Olfactory Event-Related Potentials (OERPs) are a valid electrophysiological technique for the study of olfactory system. This method allows to observe changes in olfactory function in an objective way. Indeed, it is independent from patients' response bias. OERP presence is a strong indicator of good olfactory function; conversely, the OERP absence suggests an olfactory loss.

OERPs are the result of sequential activation of different brain areas that begins from olfactory bulbs and tracts and involves the orbitofrontal and insular cortices, along with rostrum-medial regions of the temporal lobe [29]. The trasmission of olfactory sensory input travels from the olfactory neuroepithelium located into the nasal cavities towards the olfactory bulbs through the first cranial nerves, which here makes contact with the second order neurons (dendrites of mitral and tufted cells within glomeruli). From here, the postsynaptic fibers that form the olfactory tracts project to the primary olfactory areas, which comprise the anterior olfactory nucleus, tenia tecta, olfactory tubercole, piriform cortex, amygdale, anterior cortical amygdaloid nucleus, periamygdaloid and entorhinal cortices. The piriform cortex is connected to thalamus, hypothalamus and orbitofrontal cortex (OFC), and the entorhinal cortex is connected to hippocampus. The thalamus has connections towards secondary olfactory areas, as the OFC and insular cortex [30].

The OERPs consist of a large negative component, called N1, followed by a large positive component, called P2. Three scalp electrodes placed along the midline (Fz, Cz, and Pz) allow to identify the relative cortical fronto-centro-parietal regions activated by olfactory stimuli and then to detect the OERP topography. Indeed, N1 and P2 components have maximal amplitudes over the Cz and Pz positions [31]. Other components, P1 and N2, are often undetectable. The early OERP components (N1 and P1) reflect the exogenous cortical activity related to sensory input detection and primary sensory processing. On the other hand, the later OERP components, such as P2, reflect endogenous cortical activity related to secondary cognitive processing [32], [33]. Latency and amplitude are the main parameters of OERP components. Latency of N1 and P2 components is a measure of the time required for sensory and cognitive processing of odor stimuli, respectively. Amplitude reflects the significance of the stimulus and its amount of information [33]. The latency of P2 has reached high reliability and is observed approximately from 530 to 800 ms after stimulus onset. The amplitude of N1-P2 is observed approximately between 4 and 20 µv [31].

Currently, few published studies used an objective method, such as OERPs, to evaluate the olfactory function in MS [22], [23].

The purpose of our study is to evaluate the olfactory function in a group of Relapsing Remitting (RR) MS patients by using OERP technique, in order to verify objectively if the olfactory pathways are involved in neuroinflammatory and/or neurodegenerative impairment.

## Materials and Methods

### Study population

Thirty patients (19 females and 11 males) with diagnosis of RRMS according to the revised McDonald criteria [34] (mean age of 36.03±6.96 years and mean Expanded Disability Status Scale (EDSS) score of 2.08±1.07) and 30 age, sex and smoking–habit matched health controls, without neurological or psychiatric disorders, (18 females and 12 males; mean age of 35.83±8.74 years) were recruited from June to October 2013 at IRCCS Centro Neurolesi “Bonino-Pulejo” of Messina, (Italy). The disease duration ranged from 2 to 13 years with a mean duration of 5.87±3.29 years. A more detailed description of patients' and controls' characteristics is showed in Table 1.

**Table 1:** Description of patients' and normal control subjects' characteristics.

	MS patients			Normal controls		
	Females	Males	All	Females	Males	All
**Participants**	19 (63.33%)	11 (36.67%)	30 (100%)	18 (60%)	12 (40%)	30 (100%)
**Age** (mean ± SD)	35.21±8.20	37.45±4.04	36.03±6.96	33.55±9.39	39.25±6.62	35.83±8.74
**EDSS** (mean±SD)	2.13±1.21	2.00±0.84	2.08±1.07			
**DD** (mean±SD)	5.31±3.04	6.82±3.63	5.87±3.29			
**DMT**						
None	5 (16.67%)	4 (13.33%	9 (30%)			
Copaxone	1 (3.33%)	2 (6.67%)	3 (10%)			
Avonex	3 (10%)	1 (3.33%)	4 (13.33%)			
Rebif 22	2 (6.67%)	0 (0%)	2 (6.67%)			
Rebif 44	2 (6.67%	2(6.67%)	4 (13.34%)			
Gylenia	1 (3.33%)	0 (0%)	1 (3.33%)			
Tysabri	3 (10%)	1 (3.33%)	4 (13.33%)			
Extavia	2 (6.67%)	1 (3.33%)	3 (10%)			
**N1** (Cz) presences	16 (53.33%)	7 (23.33%)	23 (76.66%)	18 (60%)	12 (40%)	30 (100%)
Latencies (mean ± SD)	632.69±13.03	648.14±38.12	637.39±23.77	630.39±18.96	641.33±19.42	634.7±19.59
**P2** (Cz)presences	16 (53.33%)	7 (23.33%)	23 (76.66%)	18 (60%)	12 (40%)	30 (100%)
Latencies (mean ± SD)	720.44±16.80	743.00±11.59	727.30±18.49	711.67±20.91	728.58±20.17	718.43±21.95
Amplitudes (mean ± SD)	4.50±1.99	3.64±1.03	4.24±1.77	7.28±1.82	6.57±1.77	6.99±1.81

DD = Disease Duration; DMT = Disease Modifying Therapy; SD = Standard Deviation; EDSS = Expanded Disability Status Scale.

Latency values are in ms. Amplitude values are in µV.

A careful medical history was obtained from all participants in order to exclude diseases of nasal and paranasal cavities or other causes of smell impairment. An otorhinolaryngoiatric evaluation, by using upper airway rhinoscopy, assured the patency of nasal cavities and excluded anatomic abnormalities. We excluded the subjects treated with drugs that may affect olfactory function and/or OERP recording such as antispasmodics, antidepressants, hypnotic-sedatives and steroids. For this reason, we excluded patients who have had MS relapses treated with steroids in 3 months prior to enrollment. Other exclusion criteria were pregnancy or lactation. Relationships between OERP results and patients' clinical characteristics, such as gender, disability status (EDSS score), disease-modifying therapy (DMT), and disease duration, were evaluated.

### Olfactory evaluation

All subjects underwent an OERP examination to evaluate their olfactory function. A selective and controlled stimulation of the olfactory system to elicit the OERPs was achieved by a computer-controlled Olfactometer (Olfactometer OM2S - Burghart, Medical Instruments), linked directly with an electroencephalograph (Micromed Brain Quick 32 Ch) [35]. Olfactometer is a complex instrument which generates olfactory stimuli of rapid onset and precisely controlled in terms of time, duration and intensity, without inducing the simultaneous activation of different sensory systems (tactile, termica). During the recording two odorants were presented: phenyl ethyl alcohol (PEA, 40% v/v; Labochem Science S.r.l., Italy) and H_2_S/N_2_ (4 ppm; Rivoira, Italy). These odorants, used in appropriate concentrations, do not give any problems of toxicity and trigeminal activation. The use of the Olfactometer allows the elicitation of OERP components more easily than other methods, because the odorants do not mix and their concentration remains constant. The flow of air (8 l/min) that carries the odorants had constant temperature (36.5°C) and humidity (80%) to inhibit irritation of the nasal mucosa. The subjects did not present problems or side effects.

Subjects were asked to breath normally through their mouth. A constant level of vigilance were maintained by asking subjects to avoid eyes blinking. The teflon outlet nose (4 mm lumen tube) piece was placed in the nasal vestibulum. Stimulation was presented while subjects were lying down in a well-ventilated room. A succession of 40 randomized olfactory stimuli was presented in two blocks of 20 stimuli, alternating right and left nostril. The duration of each stimulus was 200 ms and time between stimuli (interstimulus interval, ISI) was 40 s. The change of nostril was made during ISI between twentieth and twenty-first stimulus, without interruption of the electroencephalographic (EEG) recording. EEG was recorded from three scalp electrodes placed along the midline (Fz, Cz, and Pz positions of the 10–20 International System). The references electrode was placed on the earlobe (A2) and the ground on the forehead [31]. Eye movements and blinks (electro-oculogram) were monitored by an electrode above the right eyebrow. Other muscle artifacts were monitored and discarded. The data were filtered with a band-pass 0.01–30 Hz and a notch filter was used. The EEG activity was averaged from 500 ms in the pre-stimulus period until 2000 ms in the post-stimulus period. The OERPs were obtained by averaging of artifact-free EEG epochs. The latencies were measured to the first negative peak (N1) and to the second positive peak (P2). Amplitude was measured from the peak of N1 to that of P2. OERPs were considered absent when it has not been possible to identify clear responses from background noise in an artifact free recording. The subjects who showed N1 and P2 wave presence, with normal latency and amplitude, have been considered normosmic. Conversely, the absence of N1 and P2 waves indicated a severe olfactory dysfunction. Finally, N1 and P2 wave presence with an alteration in latency and/or amplitude has been considered as a condition of slight alteration of smell (borderline).

### Statistical analysis

Statistical analysis was performed by using the 2.15.3 version of the open-source software R.

Wilcoxon rank sum test, independent Student's t-test, and X^2^ test were used to compare patient and control groups where appropriate.

In order to assess the statistical relationship between presence/absence of OERPs and disability degree, the EDSS score was first converted into a dichotomous variable according to its mean value. Then, the Fisher's exact test on the contingence table was applied.

The Kruskall-Wallis test was performed to compare the different therapeutic treatments, considering three categories: none therapy, first-line drug administered (i.e. Copaxone, Avonex, Rebif 22, Rebif 44, Extavia), second-line drug administered (i.e. Tysabri, Gylenia). Linear correlations between variables were computed by Pearson's coefficient, or the point-biserial correlation coefficient when one variable was dichotomous. For all statistical tests, a p<0.05 was considered as significance level.

### Ethical considerations

The study was conducted in accordance with the Declaration of Helsinki and was approved by Ethic Committee of IRCCS Centro Neurolesi “Bonino-Pulejo”; all subjects gave written informed consent before any study-related procedures were performed.

## Results

Table 1 summarizes the demographic and clinical characteristics of patients and controls, and the main parameters of OERP components.

**Figure 1:**
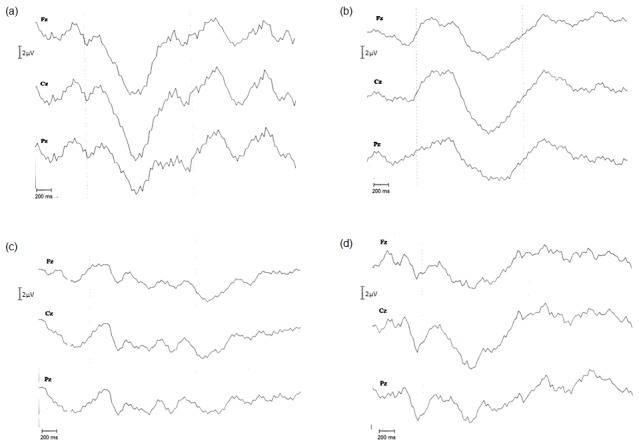
Averaged OERP traces. Filter band-pass 0.01–30 Hz. EEG averaging from 500 ms pre-stimulus to 2000 ms post-stimulus. (a) OERPs of healthy control subject; (b) OERPs of MS patient with normal latency and amplitude; (c) OERPs of MS patient without olfactory responses; (d) OERPs of MS patient with reduced N1-P2 amplitude and normal latency.

**Figure 2:**
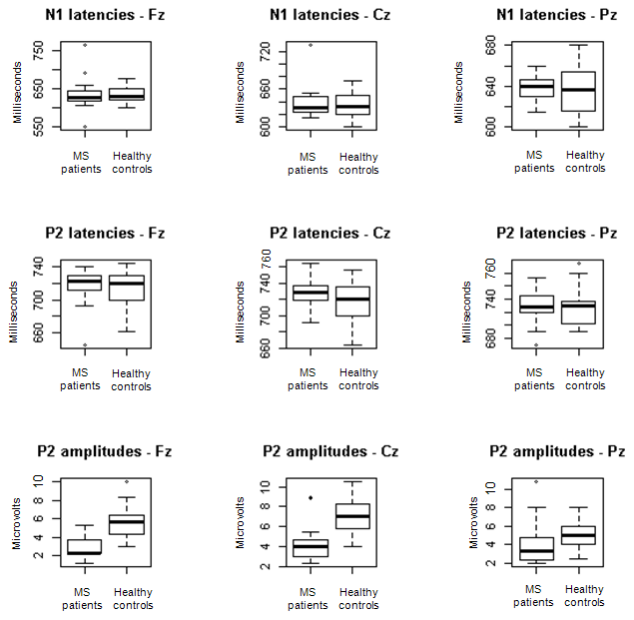
Box-plots comparing the parameters of OERP components of MS patients and healthy controls on Fz, Cz, Pz electrodes. Box-plots represent the distribution of latency and amplitude of N1 and P2 components for both MS patients and controls in Fz, Cz, Pz canals. Significant differences emerged only in P2 amplitude values, which were lower in MS patients than in controls (Fz: p<0.0001; Cz: p<0.0001; Pz: p<0.001).

All control subjects had amplitude values in the normal range [31]. Seven of 30 MS patients (3 women and 4 men) did not show OERP presence (Figure 1c). The mean age of such subgroup was slightly higher (37.71±4.96 years) than in the remaining 23 patients (35.52±7.49 years), but in a no statistically significant way (p = 0.6229). The comparisons of OERP parameters between the remaining 23 patients and the control group showed no significant differences in terms of latency of N1 and P2 components. On the contrary, very significant differences for amplitude values in each canal were observed (Fz: p<0.0001; Cz: p<0.0001; Fz: p<0.001) (Figure 2). The highest difference was observed for Fz, for which it would seem that MS women suffered of a slightly more significant reduction of N1-P2 amplitude than men (p<0.001 and p<0.01, respectively) who, instead, presented a higher difference of Pz (p = 0.0044) than women (p = 0.0159). In the light of MS group response reduction only in amplitude parameter, we also compared the mean amplitudes (computed on Fz, Cz and Pz for any subject) between two groups by considering a reference value of 3.67 µV, which was the minimum value obtained from control subjects (Table 2). Sixteen of 23 of MS patients (subjects who showed the OERP presence) had a mean value of amplitude lower than control group. (Figure 1a and 1d). In fact, we observed a very significant difference (p<0.01) by comparing mean amplitudes of MS patients with controls. The remaining 7 MS patients showed normal OERP amplitude (Figure 1b).

**Table 2:** Mean OERP values computed on Fz, Cz, Pz for each MS patient and normal control.

MS patients			Normal controls		
N1 latency (mean of Fz.Cz.Pz)	P2 latency (mean of Fz.Cz.Pz)	P2 amplitude (mean of Fz.Cz.Pz)	N1 latency (mean of Fz.Cz.Pz)	P2 latency (mean of Fz.Cz.Pz)	P2 amplitude (mean of Fz.Cz.Pz)
617.67	719.33	1.98	608.67	705.33	8.07
655.67	716.00	3.20	622.67	720.67	6.63
603.33	699.00	4.65	631.33	672.00	5.33
626.00	684.67	3.83	619.00	694.67	4.80
651.67	723.33	8.37	665.67	746.33	4.87
644.00	734.33	3.00	645.67	718.67	4.93
626.67	725.00	2.17	646.33	690.33	5.27
629.00	711.00	4.53	647.67	744.67	6.90
628.00	708.67	3.57	663.00	751.33	6.33
622.50	710.00	2.15	641.67	709.33	4.43
642.33	720.00	3.63	644.33	750.00	6.27
626.67	740.67	2.77	667.33	733.33	6.67
646.67	726.33	4.00	655.00	740.67	7.07
641.00	737.00	2.00	620.00	723.33	7.40
627.33	740.00	3.03	644.33	728.00	4.83
685.33	727.33	2.37	664.67	723.00	6.70
670.33	729.33	2.50	637.67	700.00	6.53
613.00	734.67	3.57	667.33	762.67	3.67
641.67	730.33	5.17	630.67	698.33	6.27
621.33	720.67	7.30	636.67	691.67	5.90
643.33	745.00	2.97	627.00	700.00	8.60
625.33	731.67	3.10	618.33	705.00	4.33
656.33	752.67	3.33	610.00	731.67	4.33
-	-	-	613.33	690.00	4.83
-	-	-	604.67	730.00	5.90
-	-	-	616.67	730.00	5.27
-	-	-	606.67	713.33	9.33
-	-	-	615.00	721.67	4.43
-	-	-	653.33	738.67	6.07
-	-	-	630.00	728.33	6.33

Latency values are in ms. Amplitude values are in µV. Minimum and maximum values are underlined.

No significant difference in amplitude and latency values of OERP components between the 23 MS patients and the 30 controls depending on gender was found (X^2^ = 0.18, p = 0.67). A positive correlation between gender and P2 latencies in MS group (r = 0.5742, p<0.01) was found. Comparing OERPs of MS patients with gender, we observed a significant reduction of P2 latencies on Cz (p<0.01) and Pz (p = 0.0223) positions in the MS female group. In addition, an association between EDSS and presence/absence of OERPs was noted (p = 0.0433). From these results it is emerged a negative correlation between the presence/absence of OERPs and the disease duration (r = −0.36409, p = 0.04793): a longer disease duration was related to a higher probability of recording OERP absence. We did not observe significant differences in OERPs of MS patients when compared to DMTs.

## Discussion

This study evaluated the olfactory function by using OERPs in a group of RRMS patients. Unbiased olfactory dysfunction of different severity was found. In particular, a strong impairment of olfactory function was found in 7 of 30 MS patients (23%), which no showed OERPs (Figure 1c), while 23 MS patients showed OERP responses. Of these latter, 16 (69.56%) had a marked reduction in N1-P2 amplitude, but normal latency (borderline olfactory function) (Figure 1d), and the remaining 7 patients had normal latency and amplitude of N1 and P2 components (Figure 1b).

Hawkes et al. [22] reported a OERP study where the 25% of 45 MS patients showed an impaired smell function. In particular, a statistically significant increase of N1 and P2 latency and decrease of amplitude for MS patients were found. In our study, 16 MS patients with borderline smell function reported only a significant reduction of amplitude, but not an increase of latency. These different results could be due to clinical heterogeneity of patients and to experimental conditions in which the studies have been performed. Dahlslett et al. [23] reported a study performed on 21 MS patients where the 23.8% showed hyposmia. In this study, the hyposmia or normosmia condition was based on the detection of olfactory potentials in one side or both, respectively. In our study, hyposmia diagnosis required an alteration in both latency and amplitude of main OERP components, independently from the stimulated side. A trend towards reduced P2 latency in MS female group found in our study confirms how reported from literature in healthy subjects. Indeed, several studies reported that OERPs elicited in women subjects are of shorter latency and greater amplitude than in men, suggesting an hormonal modulation of the sense of smell and sex-related differences in high levels of neural processing of odors [31], [33], [36]. In 7 patients the absence of OERPs was associated with higher EDSS scores and longer disease duration than the other 23 patients. This result corroborates how is emerged from previous studies, in which the olfactory dysfunction correlated positively with disability level [8], [9], [23].

The neuro-inflammatory processes in MS involve either the cerebral white matter, causing demyelination, and the grey matter, leading to axonal damage. In addition to the inflammation, the widespread neuronal degeneration is currently considered a central component of MS pathology [37]. Whether neurodegeneration occurs as a consequence of the inflammatory attack (secondary neurodegeneration) or as a distinct phenomenon (primary neurodegeneration) is a matter of controversy. Components of the immune/inflammatory response, such as lymphocytes, pro-inflammatory cytokines and activated macrophages, are found in close proximity to degenerating neurons, suggesting that neurodegeneration is secondary to the inflammation and that it may be considered as an underlying cause for permanent and progressive disability in MS [37], [38]. On the other hand, there is evidence that axonal transection and neuronal damage occur early supporting a primary neurodegenerative course [39]. Immune-mediated inflammation may affect the olfactory pathways. In fact, MS plaques have been found in the olfactory tracts and bulbs of MS patients [40]. Moreover, it is likely that, similar to the initial frequent involvement of the optic nerve in MS, neurodegeneration may occur in the myelin-free olfactory nerves in the early stages of disease. Several studies reported that in MS there is a relationship between smell loss and lesion load in the inferior frontal- and temporal-lobe regions, the areas involved in processing of olfactory stimuli [9], [17], [20], [21].

Our findings showed a clear correlation of olfactory dysfunction with disability degree and disease duration. This suggests that the abnormal olfactory function found in 7 MS patients could be associated with an inflammatory lesion load and also with a neurodegenerative process of olfactory networks. Conversely, the borderline olfactory function found in 16 MS patients could be associated only with a primary neurodegenerative course not yet engaging demyelination of olfactory networks. Indeed, an inflammatory demyelination of the olfactory pathways would lead to reduced conduction and inhibition in the transmission of electrophysiological impulses, and thus to a delay in latency, which has not been found in our study.

It could be postulated that the heterogeneity of olfactory dysfunction found in our patients is due to different demyelination/neurodegeneration severity in different olfactory structures. Indeed, the 7 MS patients, who did not show OERPs, could have a severe impairment of central olfactory pathways (bulbs and traits) and cortical areas. On the other hand, 16 MS patients with borderline olfactory function could have a slight impairment of olfactory central networks and/or of peripheral structures (cranial nerves I). These results could be confirmed by future MRI study, in order to analyze lesion load, plaque distribution and axonal damage in olfactory structures of MS patients in relation to degree of olfactory deficit and disability.

The MS patients present different symptoms related to different temporal-spatial distribution of the lesions. However, some symptoms are more frequent than others, such as motor impairment, dizziness, visual deficits, and urinary disorder, and some of these are more disabling and with a higher impact on quality of life than a possible impairment of smell. In fact, smell reduction is a symptom frequently ignored by patients and the enrolled subjects did not report olfactory problems. The lack of awareness of olfactory deficit in MS patients could lead to an underestimation of this disorder, which instead may be indicative of subclinical neurodegenerative process.

The close relationship between MS and olfactory dysfunction has been confirmed by several studies performed by using psychophysical tests. However, these results must be refined from all the variables that can affect the subjective tests such as the cultural factors of examined subjects, the clinical findings, mood and cognitive functions. Despite the great difficulty of OERP elicitation, which hinders their frequent use in clinical practice, in recent years this electrophysiological technique showed an important development, especially in the diagnosis of neurodegenerative diseases. In addition, to date, it reached high reliability and represents an objective method for discriminating the reduced olfactory perception.

According to our findings, we believe that the olfactory examination may be an objective marker of neuroinflammatory or neurodegenerative process in MS. However, given the rather small sample of MS patients we considered, the results must be interpreted with caution. To achieve a confirmation of results the study should be extended to a large population. Since longitudinal studies have not been still performed, they could give new information about the changes of subjects' OERPs over time and could help to monitor the possible associations among smell impairment and disease progression.
